# Decrease in AQP4 expression level in atrophied skeletal muscles with innervation

**DOI:** 10.14814/phy2.14856

**Published:** 2021-05-15

**Authors:** Minenori Ishido, Tomoya Yoshikado

**Affiliations:** ^1^ Division of Human Sciences Faculty of Engineering Section for Health‐Related Physical Education Osaka Institute of Technology Osaka Japan; ^2^ Graduate Course in Applied Chemistry, Environmental and Biomedical Engineering Osaka Institute of Technology Osaka Japan

**Keywords:** AQP4, disuse muscle atrophy, immobilization, skeletal muscle

## Abstract

Functional interaction between the selective water channel AQP4 and several ion channels, such as TRPV4, NKCC1, and Na^+^/K^+^‐ATPase, closely participate to regulate osmotic homeostasis. In the skeletal muscles, the decrease in APQ4 expression due to denervation was followed by the restoration of AQP4 expression during reinnervation. These findings raised the possibility that innervation status is an essential factor to regulate AQP4 expression in the skeletal muscles. This study investigated this hypothesis using disuse muscle atrophy model with innervation. Adult female Fischer 344 rats (8 weeks of age) were randomly assigned to either control (C) or cast immobilization (IM) groups (*n* = 6 per group). Two weeks after cast immobilization, the tibialis anterior muscles of each group were removed and the expression levels of some target proteins were quantified by western blot analysis. The expression level of AQP4 significantly decreased at 2 weeks post‐immobilization (*p* < 0.05). Moreover, the expression levels of TRPV4, NKCC1, and Na^+^/K^+^‐ATPase significantly decreased at 2 weeks post‐immobilization (*p* < 0.05). This study suggested that innervation status is not always a key regulatory factor to maintain the expression of AQP4 in the skeletal muscles. Moreover, the transport of water and ions by AQP4 may be changed during immobilization‐induced muscle atrophy.

## Graphical abstract

In the skeletal muscles, the decrease in APQ4 expression due to denervation was followed by the restoration of AQP4 expression during reinnervation. These findings raised the possibility that innervation status is an essential factor to regulate AQP4 expression in skeletal muscles. The present study investigated this hypothesis using disuse muscle atrophy model with innervation. The expression level of AQP4 was significantly decreased at 2 weeks post‐immobilization. Moreover, the expression levels of TRPV4, NKCC1 and Na^+^/K^+^‐ATPase were significantly decreased at 2 weeks post‐immobilization. In conclusion, the present study suggested that innervation status is not always a key regulatory factor to maintain the expression of AQP4 in skeletal muscles. Moreover, the transport of water and ions by AQP4 may be changed during immobilization‐induced muscle atrophy.

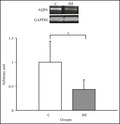

## 1 INTRODUCTION

Aquaporin (AQP) is a selective water channel with 0–12 isoforms in numerous mammalian tissues (Li & Wang, 2017; Verkman, 2005). For example, aquaporin‐4 (AQP4) is specifically expressed on the surface of the plasma membrane of myofibers in the skeletal muscles (Frigeri et al., 1998, 2001, 2004). In general, AQP4 plays an important role to regulate osmotic homeostasis by controlling the movement of water (Yukutake & Yasui, 2010).

In the skeletal muscles, the regulation of AQP4 expression was thought to be dependent on binding to α1‐syntrophin, which is a component of the dystrophin complex, on the plasma membrane of myofibers (Adams et al., 2001; Neely et al., 2001; Yokota et al., 2000). On the other hand, a significant decrease in AQP4 expression, but not in α1‐syntrophin expression, was induced in denervated skeletal muscles; therefore, α1‐syntrophin is not an essential factor for regulating AQP4 expression in skeletal muscles (Ishido & Nakamura, 2017). Thus, the key regulatory factors and regulatory mechanisms of AQP4 expression in the skeletal muscles are poorly understood.

An increase in AQP4 expression was induced in the skeletal muscles with increase in skeletal muscle activity, (Basco et al., 2013; Ishido & Nakamura, 2016) whereas AQP4 expression significantly decreased in disuse muscle atrophy induced by denervation (Ishido and Nakamura, 2017). Moreover, in skeletal muscles, the decrease in APQ4 expression due to denervation was followed by its restoration during reinnervation (Ishido & Nakamura, 2018). Therefore, these findings suggest that the innervation status by motor neurons is a key regulatory factor in regulating AQP4 expression in skeletal muscles.

It was recently demonstrated that AQP4 regulates water transport as a selective water channel, and also participates in ion transport such as for Na^+^, K^+^, Ca^2+^, Cl^−^. Three ion channels that are closely related to AQP4 are of interest. First, the transient receptor potential isoform 4 (TRPV4) is a mechanosensitive ion channel that plays important roles to control Ca^2+^ entry, thereby regulating the cell volume (Ho et al., 2012). TRPV4‐regulated Ca^2+^ transport activity is closely related to functional interaction with AQP4 (Benfenati et al., 2011; Mola et al., 2016). Second is the Na^+^‐K^+^‐2Cl^−^ cotransporter 1 (NKCC1), which regulates ion homeostasis and cell volume as an ion channel (Geck & Pfeiffer, 1985; Wong et al., 1999). Furthermore, the functional interaction between NKCC1 and AQP4 in regulating water transport and respective expression has been studied in Refs. (Yan et al., 2018; Zhang et al., 2016). Last is Na^+^/K^+^‐ATPase, a transmembrane ion pump that transports Na^+^ and K^+^ across the cell plasma membrane (Pirkmajer & Chibalin, 2016). As AQP4 functions in the regulation of Na^+^ and K^+^ transport via Na^+^/K^+^‐ATPase, the interaction between AQP4 and Na^+^/K^+^‐ATPase is important for the regulation of water, Na^+^, and K^+^ homeostasis (Illarionova et al., 2010; Strohschein et al., 2011). Although these ion channels are closely related to AQP4, the effects of the adaptive changes in the skeletal muscles to external stimulation on their expression patterns and relationships with AQP4 remain unclear.

Cast immobilization for skeletal muscles is one of the major experimental model to induce severe disuse muscle atrophy while maintaining the innervation status (Aihara et al., 2017; Booth & Kelso, 1973; Fournier et al., 1983). The present study investigated the hypothesis that innervation status by motor neurons is an essential factor in regulating AQP4 expression in skeletal muscles using a cast immobilization model. Moreover, the effects of immobilized disuse muscle atrophy on the expression of AQP4‐related ion channels, such as TRPV4, NKCC1, and Na^+^/K^+^‐ATPase, were examined.

## 2 MATERIALS AND METHODS

### 2.1 Experimental design and surgical procedure

Adult female Fischer 344 rats (8 weeks of age) were used in this study, and randomly assigned to control (C) or cast immobilization (IM) groups (*n* = 6 per group). The rats were housed in individual cages at 22°C, with a 12‐h light/dark cycle, and were provided with food and water ad libitum. All experiments were carried out after approval by the Ethics Committee on Life Sciences of Osaka Institute of Technology.

According to previous studies (Aihara et al., 2017; Booth & Kelso, 1973; Vazeille et al., 2008), rats were immobilized by arthrodesis for 2 weeks. In brief, with the rats under pentobarbital sodium anesthesia (60 mg kg^−1^ i.p.) and inhalation anesthesia with 0.7% isoflurane, the hindlimb knee and ankle joints were fixed in a neutral position with a Scotch cast (3‐J; 3M Health Care). Two weeks after cast immobilization, the rats were anesthetized and tibialis anterior muscles were removed. Muscle samples were quickly frozen in liquid nitrogen and stored at −80°C until use.

### 2.2 Western blot analysis

Sample preparation and Western blot analysis were performed according to the protocol described in a previous study (Ishido & Nakamura, 2018). In brief, the muscle samples were homogenized in 300 µl of RIPA buffer (50 mM Tris‐HCl pH 8.0, 150 mM sodium chloride, 1% NP‐40, 0.5% sodium deoxycholate, and 0.1% sodium dodecyl sulfate) (Sigma‐Aldrich) containing 1× protease inhibitor cocktail (Roche Diagnostic). After the homogenate samples were centrifuged at 12,000*g* for 10 min at 4°C, the supernatant fluids were collected. We used the BCA protein assay kit (Thermo scientific) to measure the protein concentration.

The homogenate samples (10 µg of protein) were resolved by SDS–PAGE using either a 12.5% polyacrylamide gel for AQP4, AQP1, α1‐syntrophin, and GAPDH, or a 7.5% gel for MHC‐F, TRPV4, NKCC1 and Na^+^/K^+^‐ATPase α2, and then transferred onto polyvinylidene difluoride membranes (ATTO) at 2 mA/cm^2^ for 30 min. After transfer, to block nonspecific immunoreactivity, the membranes were incubated with 20 mM Tris‐buffered saline (TBS) (pH 7.6) containing 5% normal serum and 0.1% Tween‐20 for 1 h at room temperature. The membranes were then incubated overnight at 4°C with primary antibodies. The primary antibodies were diluted in 20 mM TBS containing 5% normal serum and 0.1% Tween‐20. The primary antibodies used in the present study were as follows: goat polyclonal anti‐AQP4 (Santa Cruz biotechnology), mouse monoclonal anti‐myosin (skeletal, Fast) (MHC‐F) (Sigma‐Aldrich), rabbit polyclonal Anti‐GAPDH (internal control) (Cell Signaling Technology), rabbit polyclonal Anti‐Na^+^/K^+^ ATPase α‐2 (Merck Millipore), rabbit monoclonal Anti‐AQP1 (Abcam), rabbit polyclonal anti‐syntrophin alpha 1 (Abcam), rabbit polyclonal Anti‐TRPV4 (Proteintech), and rabbit polyclonal Anti‐NKCC1 (Abcam). Then, they were washed with 20 mM TBS containing 5% normal serum and 0.1% Tween‐20, and incubated for 1 h at room temperature with secondary antibodies. The secondary antibodies used in the present study were as follows: biotinylated anti‐goat IgG (Merck Millipore), biotinylated anti‐rabbit IgG (Invitrogen), biotinylated anti‐goat IgG (Merck Millipore), and biotinylated anti‐mouse IgG (Vector Laboratories). They were washed with 20 mM TBS containing 5% normal serum and 0.1% Tween‐20, and incubated for 1 h at room temperature with TBS containing 5% normal serum and peroxidase conjugated streptavidin horseradish (GE Healthcare). Immunoreactivity was detected by chemiluminescence using ImmunoStar Zeta (Wako). The bands were quantified by densitometric analysis using ImageJ software (ver.1.48, http://rsb.info.nih.gov/ij/). In the expression level of GAPDH, there was no significant difference between C (41004.98±12652.24 A.U.) and IM (42505.20±5424.94 A.U.) groups. So, the mean value for the control samples on each immunoblot, expressed relative to GAPDH as an internal control, was adjusted to 1.0, and each sample value was expressed relative to the adjusted mean value for the control group.

### 2.3 Statistical analysis

All data are presented as means ± SD and were analyzed using the StatView statistical‐analysis program (SAS institute Inc.). All data were tested by the Student's *t*‐test. Differences were considered significant at the confidence level of 0.05.

## 3 RESULTS

### 3.1 Effects of immobilization for 2 weeks on relative tibialis anterior muscle weights

**FIGURE 1:**
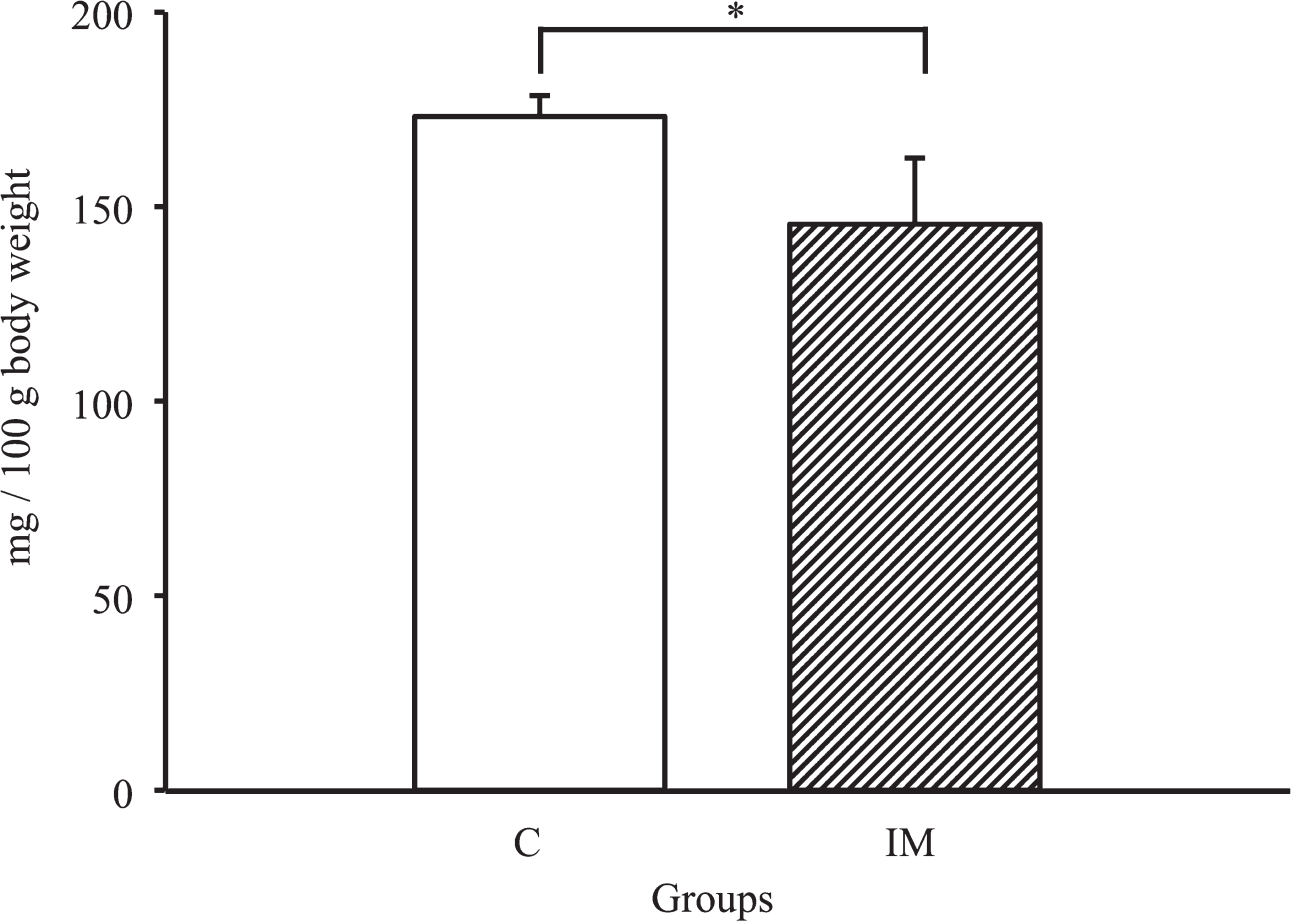
Relative tibialis anterior muscle weights in control and immobilization groups. Values are mean ± SD. Relative muscle weights are presented as mg/100 g body weight. C, control group; IM, immobilization. *p < 0.05

To assess effects of immobilization on the muscle weight, we examined the relative tibialis anterior muscle weight in each group. As shown in Figure 1, the relative tibialis anterior muscle weight in the IM group was significantly lower than that in the C group (*p* < 0.05, respectively). This suggested that muscle atrophy was significantly induced by cast immobilization for 2 weeks.

### 3.2 No change in fast myosin heavy‐chain expression after immobilization for 2 weeks

**FIGURE 2:**
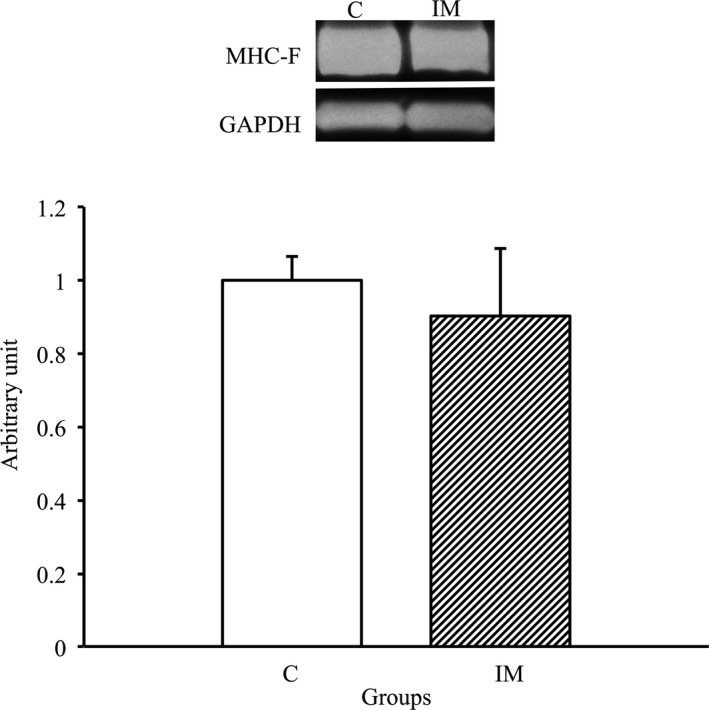
MHC‐F protein expression in tibialis anterior muscles in the control and immobilization groups. C, control group; IM, immobilization group. MHC‐F protein expression normalized to GAPDH protein expression was calculated by densitometric analysis. Values are means ± SD. Fold changes are expressed relative to the levels observed in the C group

To assess if a change in MHC‐F expression levels was induced in the skeletal muscles by immobilization for 2 weeks, we performed western blot analysis using the MHC‐F antibody. As shown in Figure 2, there was no significant difference between C and IM groups, demonstrating that MHC‐F expression was not changed in the skeletal muscles by immobilization for 2 weeks.

### 3.3 Decrease in the expression of AQP4 in the skeletal muscles due to immobilization for 2 weeks

**FIGURE 3:**
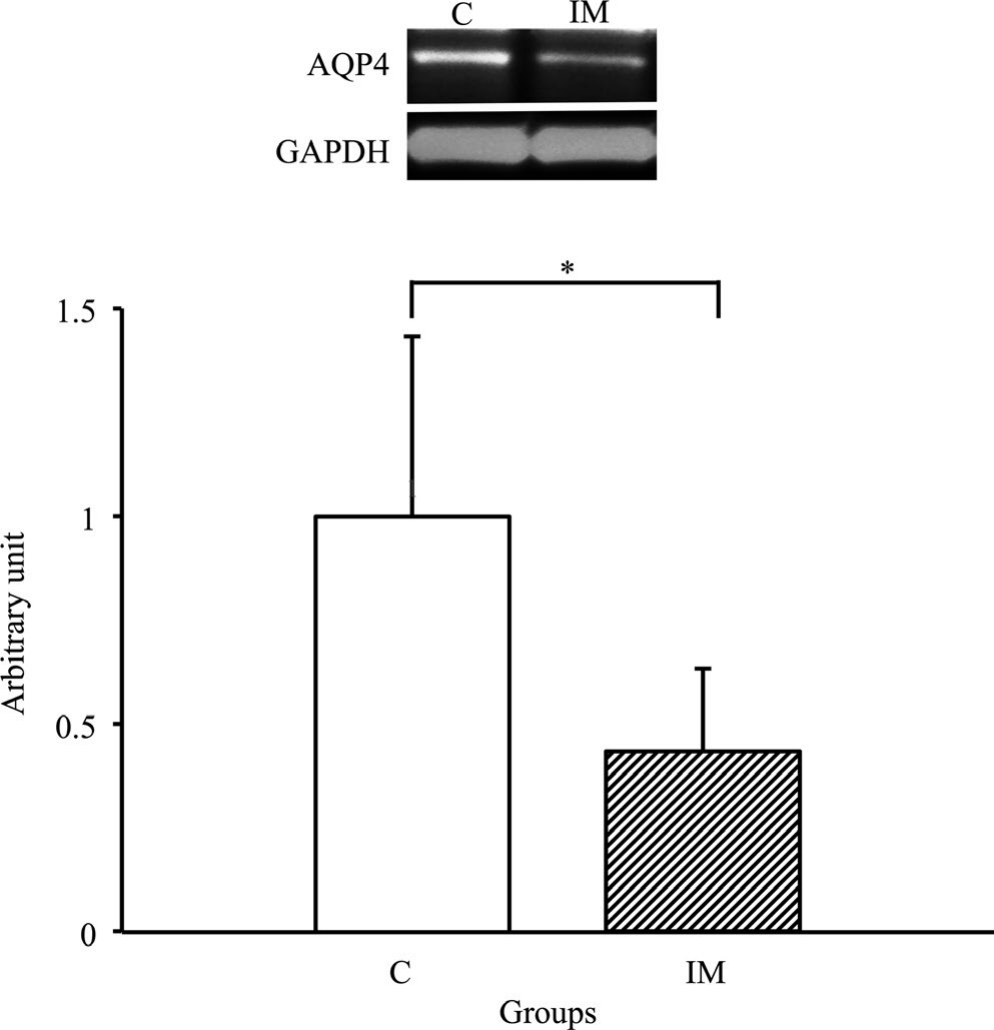
AQP4 protein expression in tibialis anterior muscles in the control and immobilization groups. C, control group. IM, immobilization group. AQP4 protein expression normalized to GAPDH protein expression was calculated by densitometric analysis. Values are means ± SD. Fold changes are expressed relative to the levels observed in the C group. *p < 0.05

We performed western blot analysis using AQP4 antibody to identify whether its expression in skeletal muscles was affected by immobilization for 2 weeks. As shown in Figure 3, the expression of AQP4 in the IM group significantly decreased compared with that in the C group (*p* < 0.05). Therefore, a significant decrease in AQP4 expression was induced in the skeletal muscles by immobilization for 2 weeks.

### 3.4 No changes in α1‐syntrophin expression after immobilization for 2 weeks

**FIGURE 4:**
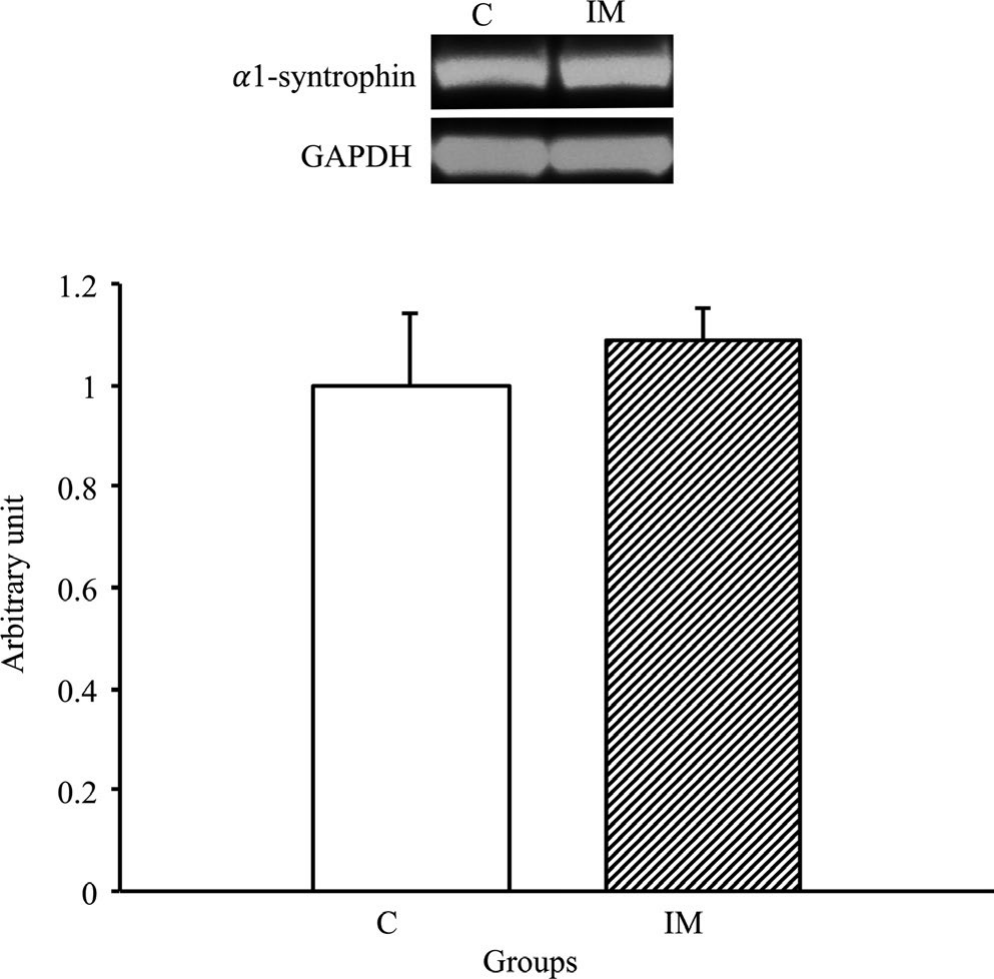
α1‐Syntrophin protein expression in tibialis anterior muscles in the control and immobilization groups. C, control group; IM, immobilization group. α1‐Syntrophin protein expression normalized to GAPDH protein expression was calculated by densitometric analysis. Values are means ± SD. Fold changes are expressed relative to the levels observed in the C group

To assess whether a change in α1‐syntrophin expression was induced in the skeletal muscles by immobilization for 2 weeks, we performed Western blot analysis using the α1‐syntrophin antibody. As a result, there was no significant difference between the C and IM groups (Figure 4). Therefore, the expression of α1‐syntrophin was not affected in the skeletal muscles by immobilization for 2 weeks.

### 3.5 Effects of immobilization for 2 weeks on the expression of TRPV4 in the skeletal muscles

**FIGURE 5:**
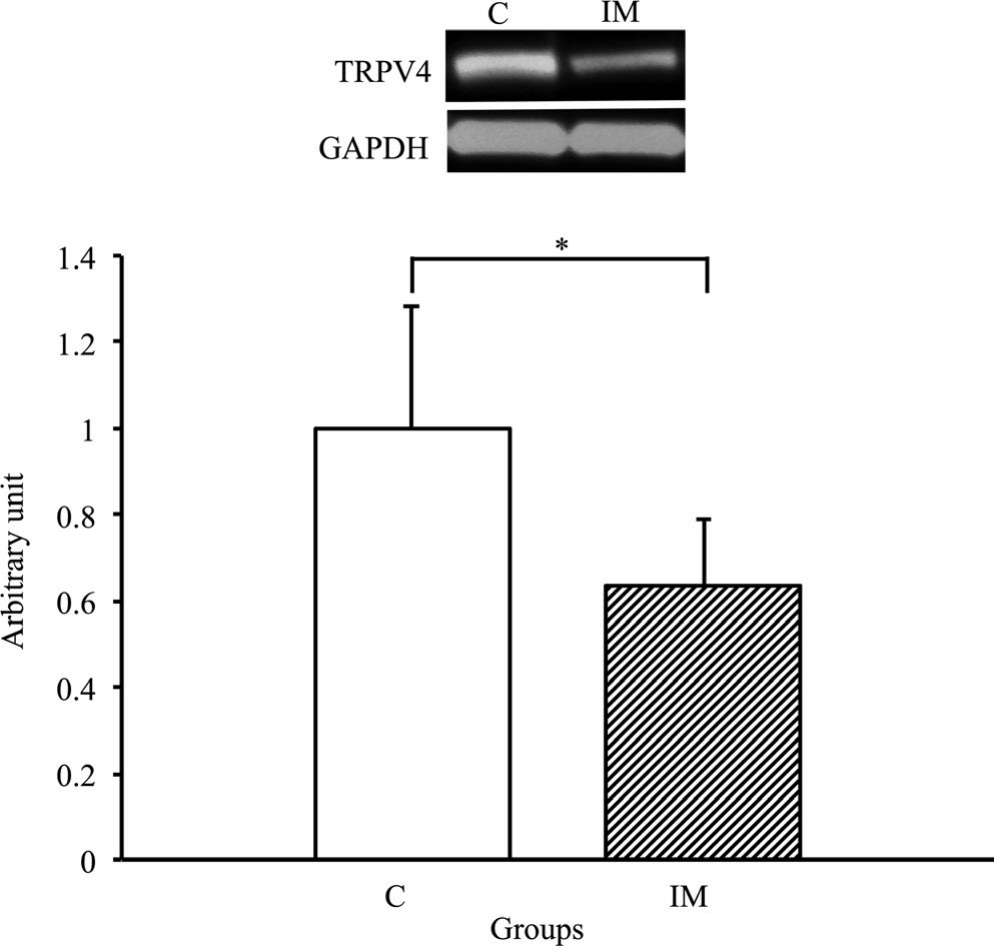
TRPV4 protein expression in tibialis anterior muscles in the control and immobilization groups. C, control group; IM, immobilization group. TRPV4 protein expression normalized to GAPDH protein expression was calculated by densitometric analysis. Values are means ± SD. Fold changes are expressed relative to the levels observed in the C group. *p < 0.05

We performed western blot analysis using TRPV4 antibody to investigate whether its expression in skeletal muscles was affected by immobilization for 2 weeks. As shown in Figure 5, the expression of TRPV4 in IM group significantly decreased compared with that in the C group (*p* < 0.05). Therefore, a significant decrease in TRPV4 expression was induced in the skeletal muscles by immobilization for 2 weeks.

### 3.6 Changes in NKCC1 expression in the skeletal muscles due to immobilization for 2 weeks

**FIGURE 6:**
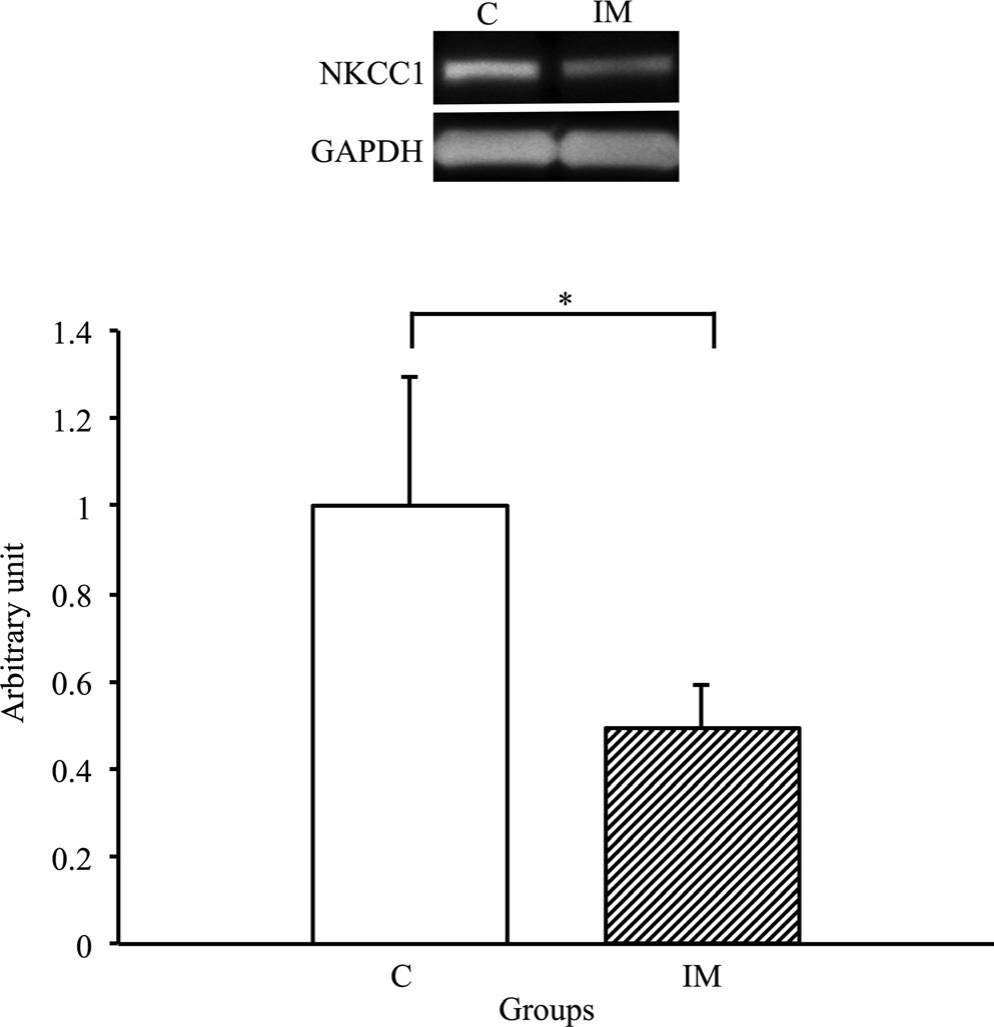
NKCC1 protein expression in tibialis anterior muscles in the control and immobilization groups. C, control group; IM, immobilization group. NKCC1 protein expression normalized to GAPDH protein expression was calculated by densitometric analysis. Values are means ± SD. Fold changes are expressed relative to the levels observed in the C group. *p < 0.05

We performed western blot analysis using NKCC1 antibody to assess whether its expression in skeletal muscles was affected by immobilization for 2 weeks. As a result, the expression of NKCC1 in the IM group significantly decreased compared with that in the C group (*p* < 0.05) (Figure 6). Therefore, a significant decrease in NKCC1 expression was induced in the skeletal muscles by immobilization for 2 weeks.

### 3.7 Decrease in Na^+^/K^+^‐ATPase α2 expression levels in the skeletal muscles due to immobilization for 2 weeks

**FIGURE 7:**
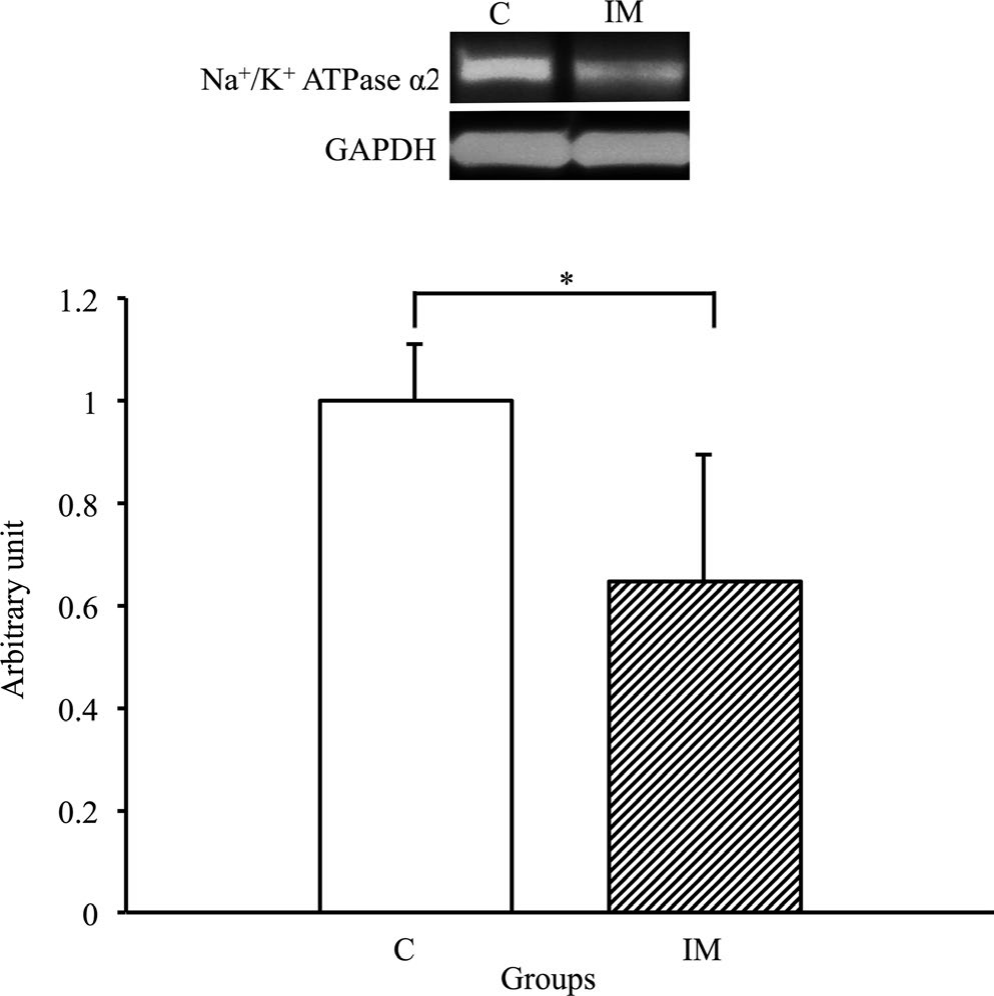
Na^+^/K^+^‐ATPase α2 protein expression in tibialis anterior muscles in the control and immobilization groups. C, control group; IM, immobilization group. Na^+^/K^+^‐ATPase α2 protein expression normalized to GAPDH protein expression was calculated by densitometric analysis. Values are means ± SD. Fold changes are expressed relative to the levels observed in the C group. *p < 0.05

To assess whether a change in Na^+^/K^+^‐ATPase α2 expression was induced in the skeletal muscles by immobilization for 2 weeks, we performed western blot analysis using the Na^+^/K^+^‐ATPase α2 antibody. As a result, the expression of Na^+^/K^+^‐ATPase α2 in the IM group significantly decreased compared with that in the C group (*p* < 0.05) (Figure 7). Therefore, a significant decrease in expression of Na^+^/K^+^‐ATPase α2 was induced in the skeletal muscles by immobilization for 2 weeks.

### 3.8 No changes in AQP1 expression after immobilization for 2 weeks

**FIGURE 8:**
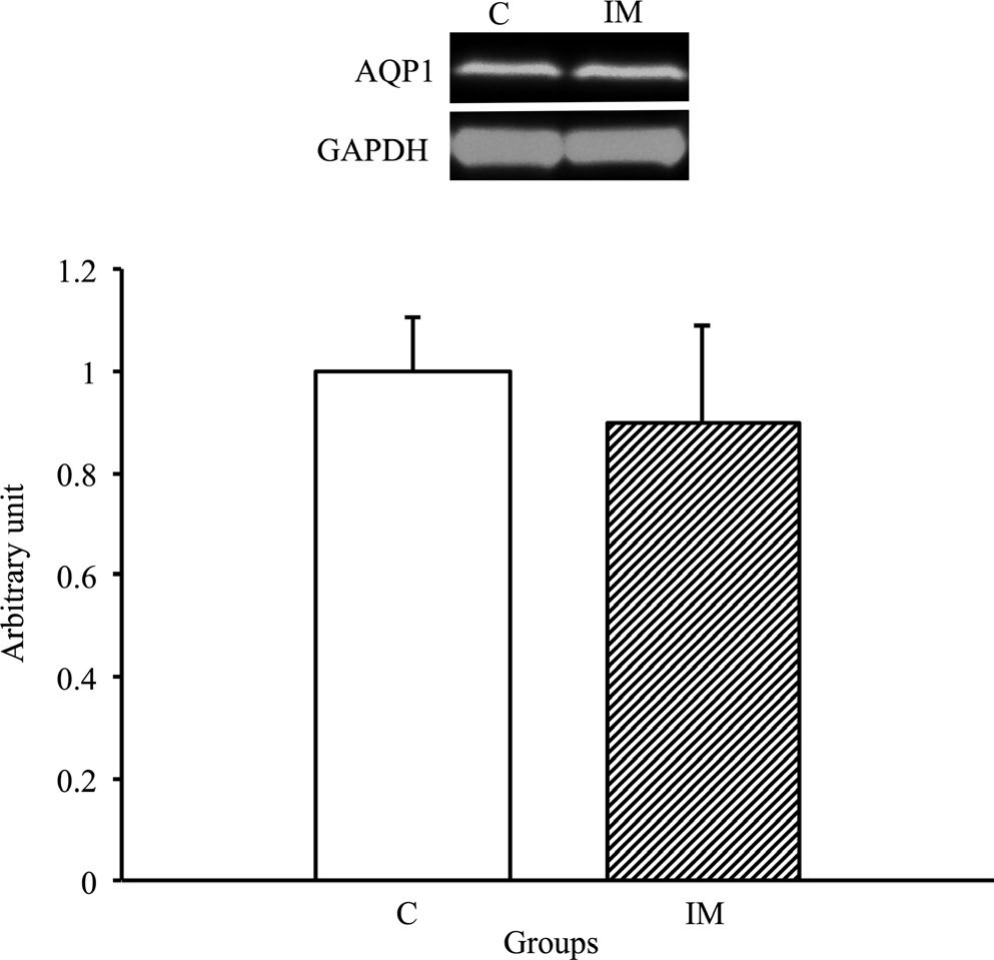
AQP1 protein expression in tibialis anterior muscles in the control and immobilization groups. C, control group; IM, immobilization group. AQP1 protein expression normalized to GAPDH protein expression was calculated by densitometric analysis. Values are means ± SD. Fold changes are expressed relative to the levels observed in the C group. *p < 0.05

To assess whether a change in AQP1 expression was induced in the skeletal muscles by immobilization for 2 weeks, we performed Western blot analysis using the AQP1 antibody. As a result, there was no significant difference between the C and IM groups (Figure 8). Therefore, the expression AQP1 was not affected in the skeletal muscles by immobilization for 2 weeks.

## 4 DISCUSSION

We recently demonstrated the possibility that innervation status by motor neurons is a major regulatory factor to maintain AQP4 expression in the skeletal muscles in vivo (Ishido & Nakamura, 2017, 2018). Therefore, in the present study, AQP4 expression levels were expected to be maintained during cast immobilization even if skeletal muscle atrophy was induced. However, a significant decrease in AQP4 expression was observed in the immobilized skeletal muscles.

In general, the expression patterns of myosin heavy chain in myofibers are considered to be involved in the regulation of AQP4 expression in the skeletal muscles, that is, APQ4 is predominantly expressed in fast‐twitch skeletal myofibers, which are abundant in fast myosin heavy chain (Frigeri et al., 1998). However, in the present study, relative MHC‐F expression levels were maintained in the skeletal muscles after 2 weeks of cast immobilization compared with control muscles. Therefore, changes in the expression of myosin heavy chain, especially fast myosin heavy chain, are unlikely to be directly involved in the decrease in AQP4 expression in skeletal muscles after 2 weeks of immobilization. Our previous study demonstrated that the decrease in AQP4 expression was prior to the decrease in MHC‐F expression in the denervated skeletal muscle (Ishido & Nakamura, 2020); therefore, the decrease in AQP4 expression may affect MHC‐F expression if the immobilization period is longer than 2 weeks.

The present study indicated that expression levels of AQP4 were significantly decreased in casted‐immobilized skeletal muscles with innervation status, and this finding was agreement with the previous study examined in the denervated skeletal muscles without innervation (Ishido & Nakamura, 2017). Therefore, these findings suggested that the innervation status is not always essential to maintain the expression of AQP4 in skeletal muscles in vivo. Although the main reason as to why AQP4 expression was reduced in denervated skeletal muscles and in immobilized skeletal muscles with innervation remains unclear, the action potential in the membrane of myofibers may be one possibility. It was previously reported that the daily activity and action potential of skeletal muscles markedly decreased after immobilization (Fournier et al., 1983; Seki et al., 2001, 2007). Moreover, the membrane potential of myofibers significantly decreased in immobilized skeletal muscles (Zemkova et al., 1990). In general, as the action potentials in myofibers are regulated by the movement of several ions, such as Na^+^, Ca^+^, K^+^ and Cl^−^, which accompany water movement (Calderon et al., 2014; Dulhunty, 2006), ion and water transport activity may be markedly decreased in immobilized skeletal muscles. Indeed, in the present study, the expression of several ion channels, such as TRPV4, NKCC1 and Na^+^/K^+^‐ATPase α2, significantly decreased in the immobilized skeletal muscles. Moreover, osmotic homeostasis and cell volume were respectively regulated by functional interaction between AQP4 and these ion channels (Chmelova et al., 2019; Illarionova et al., 2010; Jo et al., 2015; Mola et al., 2016; Strohschein et al., 2011; Yan et al., 2018; Zhang et al., 2016). Therefore, the present study suggested that water and ion transport may decrease due to the lower expression of AQP4 and related ion channels in the immobilized skeletal muscles.

Unlike AQP4, the expression of neither AQP1 nor α1‐syntrophin was changed in skeletal muscles by immobilization in the present study. These findings are similar to expression patterns previously reported for skeletal muscles exposed to denervation (Ishido & Nakamura, 2017, 2018). The present and previous studies suggested that, unlike AQP4, AQP1 and α1‐syntrophin are not intrinsically affected by muscle atrophy with or without innervation, although AQP1 and α1‐syntrophin have functional and structural interactions with AQP4. Further studies are needed to elucidate the regulatory mechanisms of AQP1 and α1‐syntrophin expression in skeletal muscles in response to external stimulation.

In conclusion, the present study revealed that AQP4 expression is significantly decreased in skeletal muscles by immobilization, suggesting that innervation status is not always a key regulatory factor to maintain the expression of AQP4 in the skeletal muscles. Furthermore, the expression TRPV4, NKCC1 and Na+/K+‐ATPase α2, which functionally interact with AQP4, also significantly decreased. Therefore, water and ion transport via AQP4 and related ion channels may decrease during immobilization‐induced disuse muscle atrophy.

## CONFLICT OF INTEREST

All authors declare no conflicts of interest.

## AUTHOR CONTRIBUTIONS

Yoshikado T contributed equally to the present study.
